# Postoperative MR Defecography following Failed STARR Procedure for Obstructive Defecation Syndrome: A Three-Centre Experience

**DOI:** 10.1155/2017/4392918

**Published:** 2017-10-03

**Authors:** Vittorio Piloni, Marco Possanzini, Mattia Bergamasco, Gianluca Santi

**Affiliations:** ^1^Radiologist Diagnostic Imaging Centre, Iniziativa Medica, Monselice, Padua, Italy; ^2^Technician Diagnostic Imaging Centre, Villa Igea Clinic, Ancona, Italy; ^3^Technician Diagnostic Imaging Centre, Iniziativa Medica, Monselice, Padua, Italy; ^4^Technician Diagnostic Imaging Centre, Studio Ronconi, Acilia, Rome, Italy

## Abstract

**Aim:**

To describe the abnormalities at MR imaging and related complaints in patients with poor outcome after STARR procedure.

**Materials and Methods:**

The medical records of 21 symptomatic patients from centre 1, 31 patients from centre 2, and 63 patients from centre 3 were reviewed with regard to findings at MR defecography and related symptoms.

**Results:**

Regardless of the centre, most relevant imaging features and related complaints were (a) impaired emptying (82.11%), related complaint ODS; (b) persistent rectocele >2 cm and intussusception (39.3%), split evacuation and digitation; (c) pelvic organ descent on straining (39.8%), prolapse sensation; (d) small neorectum and loss of contrast (32.5%), urgency and incontinence; (e) anastomotic stricture and granuloma (28.4%), pain; and (f) nonrelaxing puborectalis muscle (19.5%), tenesmus. Less frequent findings included rectal pocket formation (5.6%) and rectovaginal sinus tract (1.6%). Patients were referred to MR imaging with an average time interval of 5 ± 2, 4 ± 1, and 2 ± 1 years in the three centres, respectively, and only rarely by the same surgeon who performed the operation: 1/21 (4.8%) in centre 1, 3/39 (7.7%) in centre 2, and 9/63 (14.3%) in centre 3.

**Conclusion:**

Most surgeons involved in STARR operation with subsequent poor outcome do not rely on MR imaging.

## 1. Introduction

Stapled transanal rectal resection (STARR) for obstructed defecation was firstly described by Pescatori et al. in 1997 [1] and later reported in 1998 by Longo [2] at the Sixth World Congress of Endoscopic Surgery in Rome as an effective procedure allowing contemporary correction of rectocele and rectoanal intussusception. From the beginning, however, a fierce debate was risen between supporters and opponents of the procedure due to different data (and opinions) with regard to the functional outcome, symptoms' release, complication rate, and need for reintervention [3–11] so that at present, the real benefit of STARR has not yet been formally substantiated. Most common complaints include persistent pelvic pain, inability to evacuate completely in one or several times, increased daily frequency of stools, urgency, and need to wear a protective pad due to risk of fecal incontinence. Recently, an extensive literature review of the issue by Liu et al. [12] has highlighted that, in an attempt to reduce the failure rate, firm inclusion/exclusion preoperative criteria (still lacking) will hopefully be established as soon as possible and that occult psychosomatic components of ODS should carefully be assessed in singular cases before considering surgery. With regard to the role of diagnostic imaging, the postoperative evaluation of STARR procedure has received only limited attention in the literature, taking into account no more than the disappearance of intussuception and/or the reduction in size of rectocele, as reported by Dindo et al. [13] and Schwandner et al. [14]. Conversely, rather than relying on conventional X-ray imaging, as reported by Grassi et al. [15], magnetic resonance imaging (MRI) has been proved to be ideally suited to reveal the relationship between certain remaining symptoms and the underlying anatomical defects, particularly in case of poor outcome.

The aim of the present paper is to describe the spectrum of abnormalities and related complaints observed on MR imaging in patients with poor outcome after STARR procedure.

## 2. Subjects and Methods

All clinical and imaging series of symptomatic patients with prior STARR procedure who did not profit from the operation as anticipated were reviewed using a database system for quick retrieval and analysis of images. The diagnosis of poor outcome, usually suggested by the referring physician on the basis of medical history and clinical examination, was based on the following criteria: (a) no symptoms' release and/or recurrence of obstructed defecation at least 25% of the time after no less than six months from surgery; (b) intractable pelvic pain; (c) urgency with or without episodes of fecal incontinence; (d) any worsening or complication leading to the need for reoperation and/or intestinal diversion; and (e) persistent or recurrent symptomatic rectocele and intussusception. Patients were referred to MR defecography between July 2012 and February 2017 in three different Italian diagnostic centres (centre 1, Ancona; centre 2, Acilia, Rome; and centre 3, Monselice, Padua). The study population included twenty-one consecutive symptomatic patients (7 men, 14 women, aged 24–73 yrs, mean age 45.5 ± 2 yrs) from centre 1, observation time July 2015–January 2017; thirty-nine patients (10 men, 29 women, aged 44–69 yrs, mean age 51.4 ± 4 yrs) from centre 2, observation time July 2014–February 2017; and sixty-three patients (6 men, 57 women, aged 38–86 yrs, mean age 53.6 ± 1.8 yrs) from centre 3, observation time January 2012–February 2017. At their arrival in the radiology department, during the preliminary interview, patients were helped by the technical staff (M. P., M. B., and G. S.) to fill in a form which provided information on pre- and postoperative symptoms, including details on treatments and medical records, if any, such as 2-D or 3-D anal endosonography, anoproctoscopy, biopsy specimen, or conventional (X-ray) defecography. In particular, new onset symptoms such as pain at the anoperianal region, any passage of air and fecal material outside the vagina (women), staining episodes, or even frank incontinence with or without urgency were registered. All patients were asked to give written consent to the examination and cooperate actively to its success, having been informed on duration (average time, 24 ± 2 minutes) and need for insertion of a small catheter inside the anal canal for contrast administration.

## 3. Imaging Technique

Following uniform coaching of the technical staff in the three different diagnostic centres, all MR imaging studies were performed using the same 1.5 T horizontally oriented scanner (Philips; Achieva Sinergy model, SENSE XL TORSO coil, The Netherlands) and the same protocol. After prior rectal cleansing 2-3 hours before, patients were asked to void just 20 minutes before imaging, so as to have their bladder only half filled. In addition, to ensure collection of any rectal content without discomfort or embarrassment and to avoid contamination of the diagnostic unit, a waterproof pad was positioned under the exposed buttocks. Then, while lying on the left lateral position on the diagnostic table, retrograde rectal filling with acoustic gel was obtained via a 3.6 mm wide rubber catheter until reaching the desire to defecate. In no case, however, the amount of contrast exceeded 450 mL. Rather, should the patient experience any discomfort or involuntary loss, the injection was discontinued anticipately; in such cases, the total amount retained as well as the volume leaked were registered. After turning the patient supine, the dynamic fast images were immediately obtained with a single-slice technique on the midsagittal plane using the balance fast field echo (BBFE) pulse sequence (TR, 2.7 msec; TE, 1.3 msec; 45° flip angle; 30 mm thick section; FOV, 300 mm; 256 × 256 matrix and two averages; 1 im/0.768 sec over 90 seconds) during evacuation of the contrast. Such dynamic sequence was repeated with the use of the same parameters on the midcoronal plane before going through image acquisition on the axial plane, with sections taken horizontally during Valsalva maneuver in a steady state and the use of a multislice technique (TR, 4.1 msec; TE, 1.4 msec; flip angle, 45°; 10 mm thick section; 256 × 256 matrix and two averages; FOV, 300 mm; 2.7 sec/slice over 13 seconds). For this part of the examination, the pubic bone was taken as reference, starting image acquisition at the level of the midsymphysis (level I), then at its inferior border (level II), and finally at the point of maximal rectal descent (level III). The specific instruction for pelvic strain was the following: “take a deep inspiration so as to maintain enough air inside the chest for 15 seconds; now bend down to produce your maximal pelvic strain, starting now and holding that position without interrupting the maneuver until told to breath and relax.” After completion of the dynamic part of the examination, the pelvic anatomy was depicted at rest with images obtained on the sagittal axial and coronal planes, using the turbo spin-echo (TSE) T2-weighted pulse sequence (TR, 4630 msec; TE, 90 msec; flip angle, 90°; 4 mm thick sections, 444/310 matrix and four averages; FOV, 350 mm; acq. time, 3.37 min; total images, 35). When necessary, in case of suspected anovaginal fistula, the short tau inversion recovery (STIR) pulse sequence was employed (TR, 2768 msec; TE, 30 msec; TI, 140 msec; flip angle, 45°; 4 mm thick sections; 512 matrix and three averages; FOV, 360 mm; acq. time, 4.03 min; total images 25) choosing those planes to depict at best both the internal opening and the extent of the track.

## 4. Image Analysis

**Figure 1:**
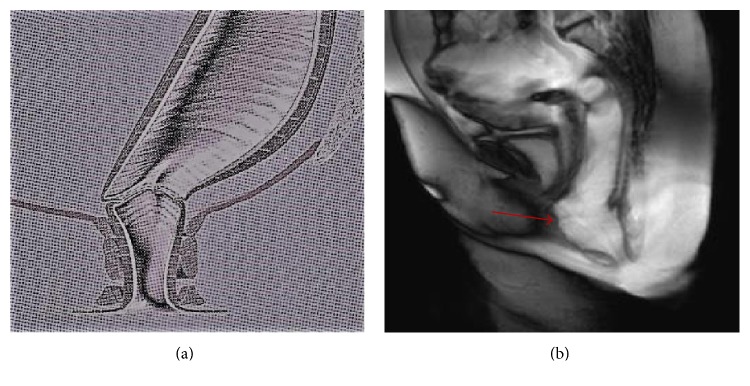
Result of STARR operation. Normal pattern: schematic drawing (a) and midsagittal BTFE pulse sequence MR image (b) taken during emptying of the intrarectal contrast; a minimal indentation is noted at the site of the anastomotic ring (arrow).

**Figure 2:**
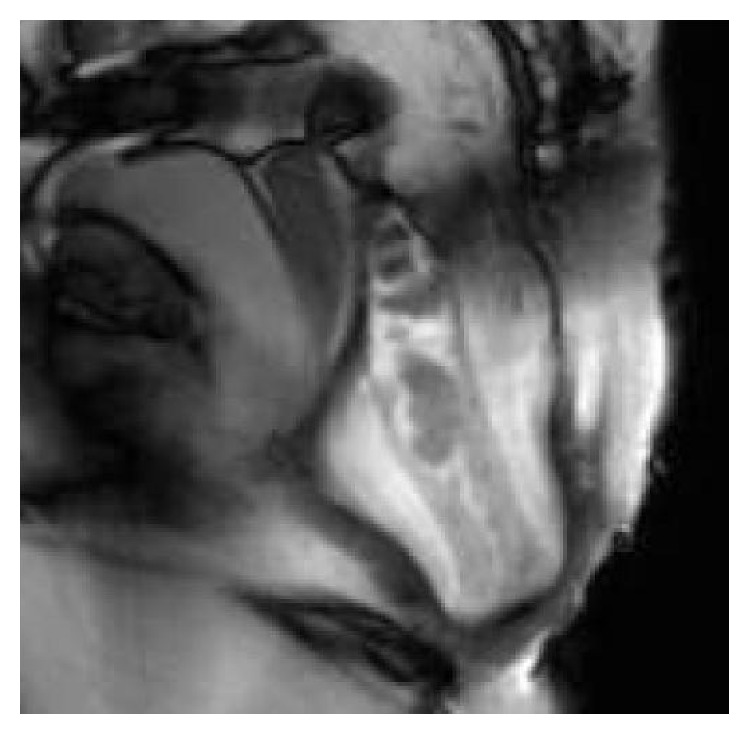
MR defecography showing persistent intussusception and complete rectal prolapse after failed STARR procedure performed 6 months earlier.

**Figure 3:**
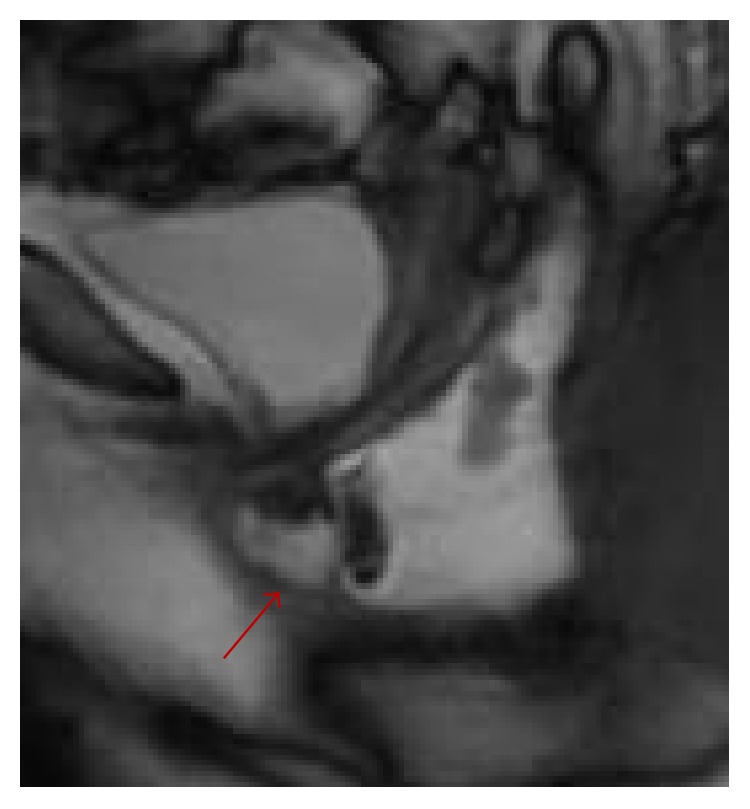
Persistent large size rectocele and contemporary depiction of a rectal pocket (arrow) developed as result of STARR operation.

All examinations were transported to a viewing station and systematically reviewed by a single radiologist (V. P.) who employed a standardized approach and used established definitions and landmarks [16–19] to analyze the postoperative anatomy, focusing the attention on the following: (a) the suture line, seen as a circular narrowing at the site of the anastomosis producing a minimal and symmetric indentation (Figure 1()), not exceeding 1 mm in depth on each side of the outer margin of the neorectum; (b) the geometrical configuration of the gut segments cranial and caudal to the suture line, including their maximal diameters at capacity, presence of asymmetry, stricture, filling defect, and extraluminal collection of contrast; (c) persistent or new abnormalities such as intussusception, rectocele, excessive descent of pelvic organs on straining, sinus track, and pocket of the rectal wall (Figures 2() and 3()); (d) and the inability to empty the contrast completely. In addition, the integrity of the internal and external anal sphincters was assessed on their real coronal and axial plane. According to the criteria described in a prior report [20], the descent of pelvic organs during straining or defecation was measured relatively to the hymen plane (woman) or to a horizontal line drawn tangent to the inferior border of the symphysis pubis (man); a descent inferior to the line was represented as a positive value, a descent above it as a negative value. A cystocele was diagnosed if the bladder base descended more than 1 cm below the reference line; similarly, a peritoneocele referred to a protrusion of the peritoneal fat below it with separation of rectovaginal septum, and an enterocele was diagnosed when the small bowel was seen to impinge on a deep Douglas pouch. Rectoanal intussusception was defined as a telescoping of the cranial segment of the rectal wall, which was pulled into itself by peristalsis, beginning as a circular infolding 6–8 cm from the anal verge, and progressively deepening to form a ring pocket that filled the entire rectal ampulla until reaching down to, into, or through the anal canal. Rectocele was defined as an anterior bulge of 2 cm or more beyond the expected line of the anterior rectal wall, while any outpouching along the posterior or lateral rectal wall protruding out through a defect of the levator ani muscle was more properly considered a perineal hernia. Finally, any abnormal outpouching of the rectal wall beyond the suture line with visible separation from the remaining part of rectum, closely resembling the shape of “dog-ear,” was defined as rectal pocket (see Figure 3()).

## 5. Data Analysis

Simple statistics of mean, standard deviation (SD), and range measurements of various parameters were calculated. Using the available data file and direct interview with patients, preoperative symptoms were compared with those after surgery and use of preoperative imaging and/or other records, when available, were analyzed. Then, the interval time between surgery and symptoms recurrence or their worsening was registered and taken as indication of failed STARR operation. In addition, the time interval between surgery and MR imaging was calculated, together with the frequency with which the surgeon performing STARR operation coincided with the referring physician of MR imaging. Finally, the type and proportion of changes seen at MR defecography relative to the corresponding most relevant daily complaints (i.e., occurring >60% of times) were registered.

## 6. Results

At dismissal, STARR operations were considered technically successful without intra- or postoperative complications in all but 8 cases (one from centre 1, four from centre 2, and three from centre 3) due to minimal complaints, including bleeding and transient pelvic pain requiring treatment by analgesics. On the other hand, no patient reported to have a comfortable evacuation pattern for more than 25% of their movement after an average time interval of 8 ± 2, 11 ± 3, and 5 ± 2 months from surgery in the three different centres, respectively (Table 1). Moreover, despite surgical correction of rectocele and intussusception and pelvic organ prolapse (percentage decrease −65% and −29%, resp.), recurrent symptoms of ODS or even their worsening increased by 22% (Table 2). However, the main de novo postoperative complaint, almost absent before surgery (2 versus 44 cases, +2.100%), was an intractable pelvic pain with or without defecation which persisted all day long, followed by tenesmus (1 versus 20 cases, +1.900%) and fecal urgency with episodes of fecal incontinence (2 versus 30 cases, +1.400%). In addition, 12 patients, compared with 2 before surgery (+500%), began developing lower urinary tract (LUT) symptoms and serious problems associated with their sexual activity. With regard to the preoperative diagnosis, it had relied on physical examination in all cases, imaging studies in 35/123 (28%), P-studies in 7/123 (6%), and anoproctoscopy in 4/123 (3%).

**Figure 4:**
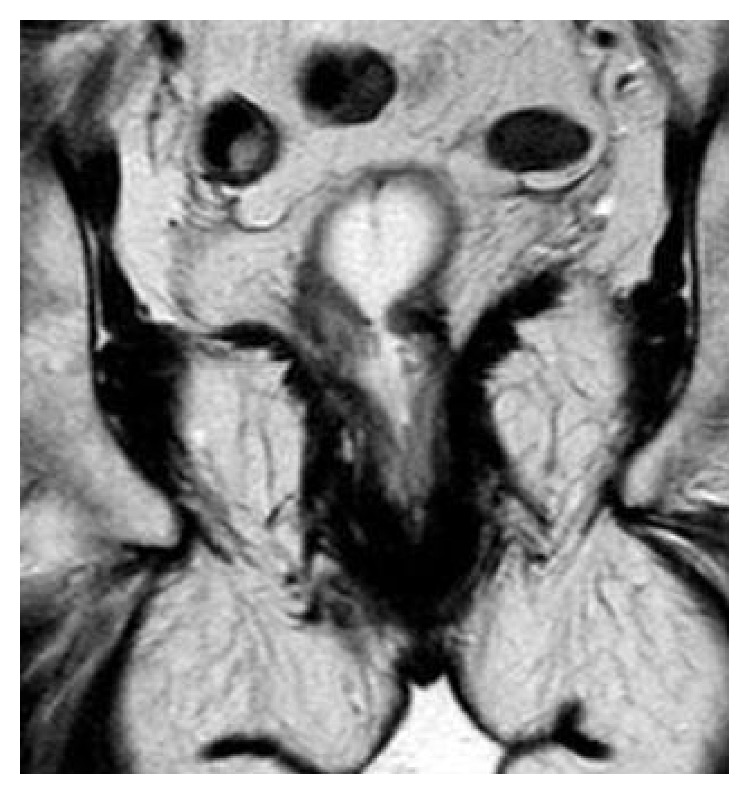
Small, uncompliant neorectum associated with significant narrowing of the anastomotic ring in a fifty-four-year men with fecal urgency and staining episodes. Compare it with that shown in Figure 1(b).

**Figure 5:**
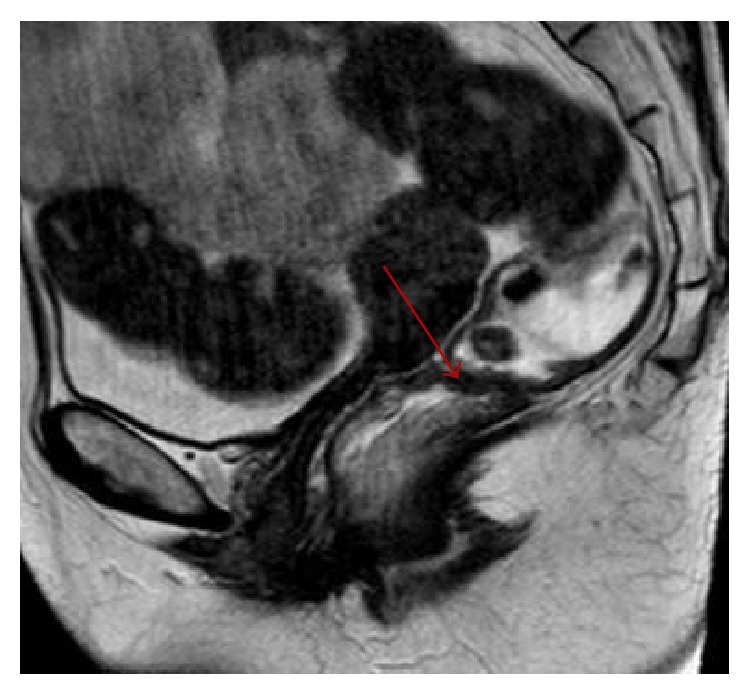
Postevacuating MR image showing thickening at the anastomotic line (arrow) and trapping of contrast in the segment cranial to it.

On postoperative MR defecography, a number of changes were discovered (Table 3), including incomplete evacuation of contrast in 101/123 cases (82.11%), persistent pelvic organ prolapse in 49/123 (39.8%), small uncompliant neorectum (Figure 4) in 40 (32.52%), stricture at the anastomotic line or around it associated with contrast retention (Figure 5) in 35 (28.45%), and persistent rectocele greater than 2 cm in 33/123 cases (26.8%). Interestingly, the frequency with which the surgeon and the referring physician of postoperative MR imaging coincided was 1/21 (4.8%) in centre 1, 3/39 (7.7%) in centre 2, and 9/63 (14.3%) in centre 3.

## 7. Discussion

Stapled transanal rectal resection (STARR) was developed two decades ago to correct obstructed defecation caused by rectocele and rectoanal intussusception. For the procedure, a circular stapling device is inserted through the anal canal into the lower rectum and kept open to include the bulk of the prolapsed mucosa inside. Then, two separate anterior and posterior rectotomies are applied to reduce the rectocele and intussusception. In case of bleeding, some stitches are applied at the suture line and the removed tissue is sent to the histology laboratory for ruling out a possible solitary rectal ulcer syndrome. Usually, the patient is discharged on the day after surgery and kept on a low-residue diet for three days, before being administered an enema and receiving the instruction to avoid straining at stool.

Most of the time, with an average operative time of 20 minutes and no major postoperative complication or pain, early return to normal daily work and social activities has been reported by various researchers since the beginning of their experience without evidence of symptom recurrence. On the other hand, contemporary experience by different groups of researchers has highlighted the development of high morbidity and failure rate associated with the procedure, including significant continence problems, intractable pain, recurrence of ODS within 2–6 months, anovaginal fistula, and pelvic sepsis, leading to need for reoperation and even to intestinal diversion. Although the use and abuse of STARR procedure remain a controversial topic and its overt superiority over conventional operations after randomized controlled trials has not been demonstrated yet, the present experience seems to give value to the latter opinion but deserves further analysis. More specifically, given the objective difficulty to obtain widespread consent by the surgeons involved in the use of the procedure to routinely refer all their patients for MRI assessment of postoperative anatomy, only those cases with poor outcome, who were consecutively referred to three different diagnostic centres from all over the country, were included in our study population. This, in turn, inevitably determined the study design aimed to deliberately bring to its extreme the effect of human (surgeon) variability, so as to restrict the analysis of the origin of poor outcome to the intrinsic value of the procedure itself. On the other hand, the real failure rate with respect to the entire population submitted to STARR operation in Italy remains unknown, and this represents an obvious limitation of the study which, nevertheless, brings some useful information to the reader. First of all, it emerged that, regardless of the expertness and reputation of the surgeon who performed the procedure—varying from the last minute coloproctologist to the most famous congress chairperson—the type of postoperative morbidity was in the range of that reported in the literature, as follows: no case of death associated with the procedure and just two cases requiring intestinal diversion with stoma construction due to rectovaginal fistula and pelvic sepsis, respectively; the latter was related to rectal perforation after rectotomy. It can be hypothesized (but not proved) that the causes of failure might concern one of the following categories: technical errors, underestimated contraindications, or ineffective treatment of the targeted lesions. However, an additional factor to consider is the emerging role of preoperative assessment as a whole. As a matter of fact, indeed, the available data demonstrate that careful selection before admission to surgery was largely deficient or lacking in the study population. This assumption is testified by the low rate of preoperative diagnostic medical records exhibited by patients at the moment of the preliminary interview. Most of the time, the indication to surgery was based on the physical examination alone without the help of imaging or any other instrumental diagnostic tool. The potential role of that behaviour in contributing to increase the risk of failure, especially when combined with underestimation of overt contraindication, such as nonrelaxing puborectalis muscle, should be highlighted. A second aspect to consider is that the same reluctancy in seeking an objective tool for the evaluation of the outcome of the procedure was confirmed by the most striking result of the present study, that is, that the postoperative MR investigation was requested by the same surgeon who performed the STARR operation only in one out of 21 (4.7%), three out of 39 (7.6%), and nine out of 63 (14.2%) cases in the three diagnostic centres, respectively. In all remaining cases, patients underwent MR defecography thanks to the referral of a different physician, after having been labeled as “technically successful” by the prior surgeon. It can be argued that, while avoiding any postoperative investigation in asymptomatic patients is easily agreeable, the same behaviour cannot be justified in case of recurrence of symptoms, or even their worsening. All the above considering the fact that today, by the combined analysis of both static and dynamic images, MRI of the pelvic floor allows radiologists to accurately identify the underlying structural defects and provides the evidence for treatment planning, reducing the risk of surgical failure, symptoms recurrence, and need for reoperation.

**Figure 6:**
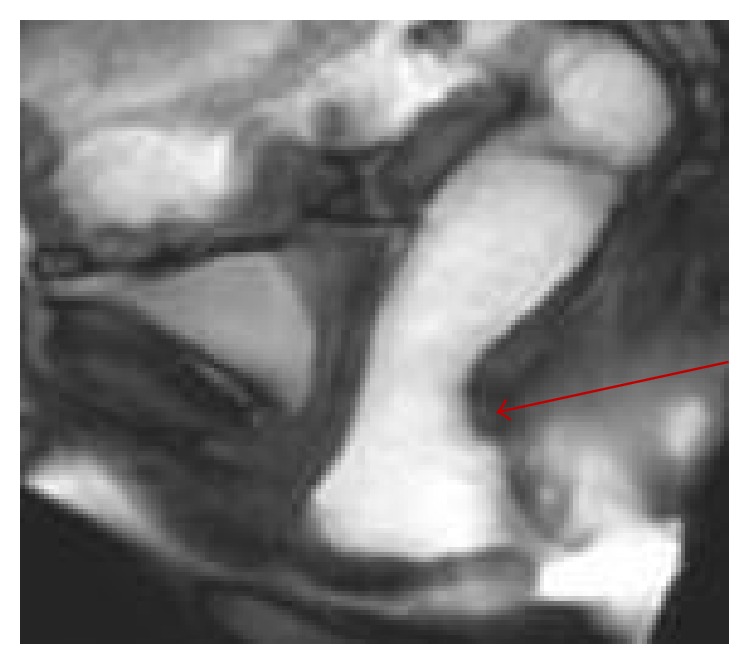
Example of wrong indication to STARR operation: postoperative MR defecography in patient with persistent nonrelaxing puborectalis muscle (arrow), responsible of recurrent rectocele and ODS.

**Figure 7:**
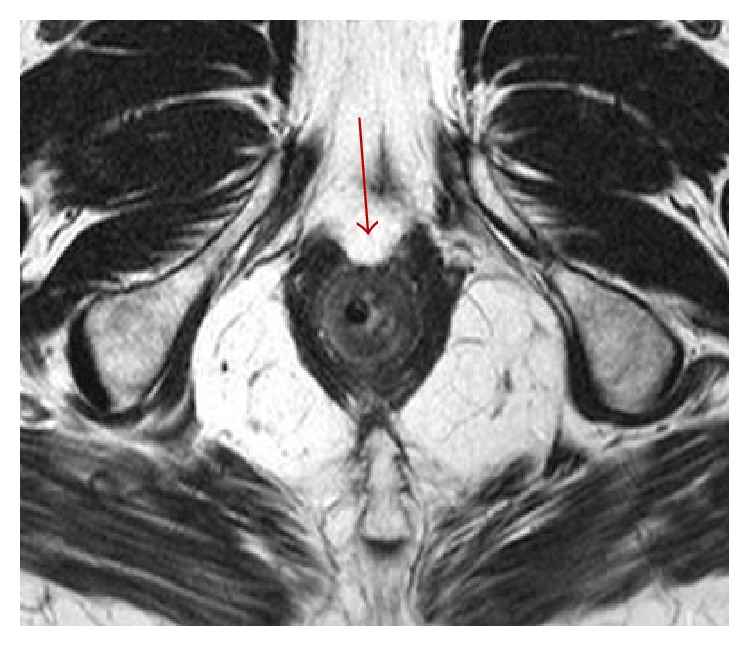
MR T2-weighted axial image of the anal sphincter complex taken perpendicular to the intra-anal marker: a focal defect (arrow) is visible from 11 to 1 (anal clock) after STARR procedure, responsible of episodes of fecal incontinence.

In our study population, the most common complaint was a recurrent obstructed defecation (82.11%), due to de novo postoperative rectoanal stricture or to underestimated preexisting abnormalities, such as nonrelaxing puborectalis muscle (Figure 6()), enterocele, peritoneocele, or pelvic organ prolapse (POP), whose correction was not taken under consideration by STARR. Occasionally however, a rectal pocket was also found by chance (5.69%) whose development can be related to the presence of two lateral bridges of residual mucosa, produced by separate anterior and posterior stapler firing unequally when performed around the circumference of the rectal wall. If sufficient in size, depending on its relationship with the outflow tract, such abnormality produces a true cul-de-sac near the staple line causing entrapment of fecal material, proctalgia, and soiling [21] and is seen to adversely affect the speed and effectiveness of contrast evacuation. On the other hand, most relevant causes of poor outcome affecting the quality of life in singular cases were (a) intractable anal pain (28.45%) at or after defecation, leading even to need for reintervention, excision of the staple line, and manual refashioning of the anastomosis. Reported causes of pain include smooth muscle incorporation in the doughnut, low placement of staples too close to the dentate line, and retained metal clips in proximity of the stapler line, producing chronic inflammatory/granulomatous reaction and thickening; (b) impaired rectal reservoir/capacity (35.52%) due to the decreased rectal size from endorectal resection. When combined with damage of the anal sphincter (Figure 7()) secondary to overstretching by the large anal dilator stapling device or the metal retractor, these changes became easily responsible for episodes of postoperative fecal urgency, soiling, and overt incontinence. How to manage postoperative complications after STARR failure remains a very complex issue indeed because of the various difficulties encountered. More particularly, in case of severe anorectal stricture not responding to anal dilatations, pelvic sepsis, or rectovaginal fistula, the only chance offered to the patients is reintervention. The same applies to recurrent intussusception and rectocele, while postoperative fecal incontinence, due to reduced rectal compliance, may improve with time and usually takes advantage from pelvic floor muscle rehabilitation programs. Conversely, injection of bulking agents within the intersphincteric space may be of some help in cases of localized lesions of the sphincteric anal complex, even though chronic pelvic pain from retained staples or pudendal nerve ending entrapment by fibrosis can be considered the real crucial point which affects the life quality of some patients after STARR.

## 8. Conclusions

Originally aimed at simply describing the spectrum of abnormalities seen on MR defecography in patients with failed STARR procedure, the present paper gives the clue for further research in the complex issue of optimal outcome following surgical reconstruction of the anorectal region. Although STARR operation can be considered a technical advance in the armamentarium of the coloproctologist, careful preoperative selection of patients remains the key to successful outcome.

Established contraindications include anismus, POP with or without enterocele, and impaired efficiency in anal closing mechanism. Today, many surgeons in Italy still have a wrong conception of the use of imaging techniques in the postoperative evaluation of STARR. The present survey shows that, even in case of poor outcome, the radiologist is only rarely involved in the search of possible causes of failure. Conversely, considering that even in expert hands, technical errors, underestimated contraindications, or ineffective treatment of the targeted abnormalities frequently occur, postoperative MR imaging is assuming a new important and expanding role today and should be considered early in the course of any adverse clinical manifestation. The present paper offers an evidence that MRI can no longer be used just to confirm the disappearance of the preexisting intussusception or the reduction of rectocele size. Rather, it provides a tool to accurately identify various changes responsible for STARR failure, most of which potentially amenable of repair.

## Figures and Tables

**Table 1:** Clinical characteristics of the study groups by diagnostic centre.

Variable	Centre 1	Centre 2	Centre 3
Sample size	21	39	63
Observation time (months)	19	26	61
Gender (F/M)	14/7	29/10	56/6
Age (yrs)			
Mean	45.5 ± 2	51.4 ± 4	53.6 ± 1.8
Range	24–73	44–69	38–86
Time interval 1 (yrs)			
Surgery-to-imaging			
Mean	5 ± 2	4 ± 1	2 ± 1
Range	3–6	2–5	1–4
Time interval 2 (months)			
Surgery-to-symptom recurrence			
Mean	8 ± 2	11 ± 3	5 ± 2
Range	2–10	1–15	3–12

**Table 2:** Comparison of pre-postoperative symptoms and use of preoperative diagnostic tools by centre in the patient population.

Variable	Centre 1		Centre 2		Centre 3		Total		Total sample statistics
	Pre	Post	Pre	Post	Pre	Post	Pre	Post	Delta post versus pre
*Symptoms*									
Sample size	21		39		63		**123**		
ODS	13	18	22	27	41	48	**76**	**93**	*22%*
Rectocele/intussusception	14	2	24	8	54	22	**92**	**32**	*−65%*
POP	6	2	4	4	11	9	**21**	**15**	*−29%*
Pain	1	9	1	12	0	23	**2**	**44**	*2.100%*
Tenesmus	0	5	0	3	1	12	**1**	**20**	*1.900%*
Urgency/incontinence	1	2	0	6	1	22	**2**	**30**	*1.400%*
LUTs/sexual	0	2	0	1	2	9	**2**	**12**	*500%*
Reintervention	n.a.	1	n.a.	2	n.a.	5	**n.a.**	**8**	*n.m.*
Stricture	n.a.	1	n.a.	2	n.a.	3	**n.a.**	**6**	*n.m.*

*Preoperative diagnostic tools*									Rate
Physical examination	21		39		63		**123**		*100%*
Imaging studies	4		11		20		**35**		*28%*
P-studies	0		3		4		**7**		*6%*
Anoproctoscopy	0		2		2		**4**		*3%*
EMG	0		0		1		**1**		*1%*

ODS: obstructed defecation syndrome; POP: pelvic organ prolapse; LUTs: lower urinary tract symptoms; n.a.: not available; n.m.: not meaningful.

**Table 3:** Diagnostic yield at MR defecography and related complaints in 123 consecutive patients with STARR failure.

MR Findings^&^	*n*°	%	Complaint^&&^
Impaired contrast emptying	101	82.11	ODS
Rectocele			
<2 cm	41	33.33	Split evacuation/digitation
>2 cm	33	26.82	
POP	49	39.83	Prolapse sensation
Uncompliant rectum	40	32.52	Urgency
Anastomotic stricture/granuloma	35	28.45	Pain
Nonrelaxing puborectalis muscle	24	19.51	Tenesmus
Intussusception	15	12.19	Incomplete evacuation
Rectal pocket	7	5.69	Fecal blockade
Anal sphincter damage/scarring	6	4.87	Incontinence
Rectovaginal fistula	2	1.62	Passage of air/discharge
Diverting colostomy	1	0.81	Prior pelvic sepsis

^&^More than one finding for patient; ^&&^occurring >60% of times.

## References

[1] Pescatori M., Favetta U., Dedola S., Orsini S. (1997). Transanal stapled excision of rectal mucosal prolapse. *Techniques in Coloproctology*.

[2] Longo A. Treatment of haemorrhoidal disease by reduction of mucosal and haemorrhoidal prolapse with a circular suturing device: a new procedure.

[3] Dodi G., Pietroletti R., Milito G., Binda G., Pescatori M. (2003). Bleeding, incontinence, pain and constipation after STARR transanal double stapling rectotomy for obstructed defecation. *Techniques in Coloproctology*.

[4] Boccasanta P., Venturi M., Stuto A., Bottini C., Caviglia A., Carriero A. (2004). Stapled transanal rectal resection for outlet obstruction: a prospective, multicentric trial. *Diseases of the Colon and Rectum*.

[5] Pescatori M., Dodi G., Salafia C., Zbar A. P. (2005). Rectovaginal fistula after double-stapled transanal rectotomy (STARR) for obstructed defaecation. *International Journal of Colorectal Disease*.

[6] Yao L., Zhong Y., Xu J., Xu M., Zhou P. (2006). Rectal stenosis after procedures for prolapse and hemorrhoids (PPH)—a report from China. *World Journal of Surgery*.

[7] Pescatori M., Gagliardi G. (2008). Postoperative complications after procedure for prolapsed hemorrhoids (PPH) and stapled transanal rectal resection (STARR) procedures. *Techniques in Coloproctology*.

[8] Gagliardi G., Pescatori M., Altomare D. F. (2008). Results, outcome predictors, and complications after stapled transanal rectal resection for obstructed defacation. *Diseases of the Colon and Rectum*.

[9] Pescatori M., Zbar A. (2009). Reinterventions after complicated or failed STARR procedure. *International Journal of Colorectal Disease*.

[10] Jayne D. G., Schwandner O., Stuto A. (2009). Stapled transanal rectal resection for obstructed defecation syndrome: one-year results of the European STARR registry. *Diseases of the Colon and Rectum*.

[11] Boccasanta P., Venturi M., Roviaro G. (2011). What is the benefit of a new stapler device in the surgical treatment of obstructed defecation? Three-year outcomes from a randomized controlled trial. *Diseases of the Colon and Rectum*.

[12] Liu W. C., Wan S. L., Yaseen S. M. (2016). Transanal surgery for obstructed defecation syndrome: literature review and a single-centre experience. *World Journal of Gastroenterology*.

[13] Dindo D., Weishaupt D., Lehmann K., Hetzer H. F. H., Clavien P. A., Hahnloser D. (2008). Clinical and morphological correlation after stapled transanal rectal resection for obstructed defecation. *Diseases of the Colon and Rectum*.

[14] Schwandner T., Hecker A., Hirschburger M., Hecker M., Kierer W., Padberg W. (2011). Does the STARR procedure change the pelvic floor: a preoperative and postoperative study with dynamic pelvic floor MRI. *Diseases of the Colon and Rectum*.

[15] Grassi R., Romano S., Micera O., Fioroni C., Boller B. (2005). Radiographic findings of post-operative double stapled trans anal rectal resection (STARR) in patients with obstructed defecation syndrome (ODS). *European Journal of Radiology*.

[16] Lienemann A., Anthuber C., Baron A., Kohz P., Reiser M. (1997). Dynamic MR colpocystorectography assessing pelvic floor descent. *European Radiology*.

[17] Comiter G. V., Vasavada S. P., Barbaric Z. L., Gousse A. E., Raz S. (1999). Grading pelvic prolapse and pelvic floor relaxation using dynamic magnetic resonance imaging. *Urology*.

[18] Piloni V., Tosi P., Vernelli M. (2013). MR-defecography in obstructed defecation syndrome (ODS): technique, diagnostic criteria and grading. *Techniques in Coloproctology*.

[19] Piloni V., Asteria C. R., Bellarosa S., Zbar A. P., Madoff R. D., Wexner S. D. (2013). Defecography. *Reconstructive Surgery of the Rectum Anus and Perineum, Vol. 2*.

[20] Piloni V., Ambroselli V., Nestola M., Piloni F. (2016). Quantification of levator ani (LA) hiatus enlargement and pelvic organs impingement on Valsalva maneuver in parous and nulliparous women with obstructed defecation syndrome (ODS): a biomechanical perspective. *Pelviperineology*.

[21] Pescatori M., Spyrou M., Cobellis L., Bottini C., Tessera G. (2006). The rectal pocket syndrome after stapled mucosectomy. *Colorectal Disease*.

